# Proof of Concept of Telemedicine-Assisted Abdominal Ultrasound Examinations to Improve the Quality of Patient Care in Rural Areas

**DOI:** 10.3390/jcm13061721

**Published:** 2024-03-17

**Authors:** Tobias Kleemann, Denise Müller, Carola Güther, Alina Duma, Awsan Mohamed, Helmut Ernst, Madlen Löbel, Robert Freund, Sven Kleemann, Sven Pannach, Rutker Stellke, Dirk Briesemann, Tina Diepelt, Ina Thomas, Viktoria Ermisch, Dimitrios Aretakis, Alexander Wree, Frank Tacke, Steffen Ortmann, Marten Schulz

**Affiliations:** 1 Department of Gastroenterology and Rheumatology, Carl-Thiem-Klinikum Cottbus, 03048 Cottbus, Germany; denise.mueller@ctk.de (D.M.); c.guether@ctk.de (C.G.); v.duma@ctk.de (A.D.); a.mohamed@ctk.de (A.M.); h.ernst@ctk.de (H.E.); 2 Department of Clinical Research, Carl-Thiem-Klinikum Cottbus, 03048 Cottbus, Germany; m.loebel@ctk.de; 3 Thiem-Research GmbH, Carl-Thiem-Klinikum Cottbus, 03048 Cottbus, Germany; r.freund@ctk.de (R.F.); s.ortmann@ctk.de (S.O.); 4 Department of Medical Informatics and Documentation, Carl-Thiem-Klinikum Cottbus, 03048 Cottbus, Germany; s.kleemann@ctk.de; 5 Department of Internal Medicine, Naemi-Wilke-Stift Guben, 03172 Guben, Germany; s.pannach@naemi-wilke-stift.de; 6 Department of Gastroenterology, Helios Klinikum Pirna, 01796 Pirna, Germany; 7 Department of Surgery, Naemi-Wilke-Stift Guben, 03172 Guben, Germany; r.stellke@naemi-wilke-stift.de (R.S.); oa-chir@naemi-wilke-stift.de (D.B.); 8 Project and Development Center, Naemi-Wilke-Stift Guben, 03172 Guben, Germany; t.diepelt@naemi-wilke-stift.de (T.D.); v.ermisch@naemi-wilke-stift.de (V.E.); 9 Department of Hepatology and Gastroenterology, Campus Charité Mitte and Campus Virchow-Klinikum, Charité—Universitätsmedizin Berlin, 10178 Berlin, Germany; dimitrios.aretakis@charite.de (D.A.); alexander.wree@charite.de (A.W.); frank.tacke@charite.de (F.T.); marten.schulz@charite.de (M.S.)

**Keywords:** abdominal ultrasound, telemedical assistance, sonography, randomized trial

## Abstract

(1) **Background:** Unclear sonographic findings without adequate specialist expertise in abdominal ultrasound (AU) may harm patients in rural areas, due to overlooked diagnoses, unnecessary additional imaging (e.g., CT scan), and/or patient transport to referral expert centers. Appropriate telemedical sonography assistance could lead to corresponding savings. (2) **Methods:** The study was designed as a randomized trial. Selected study centers performed AU with the best local expertise. Patients were selected and monitored according to the indication that they required AU. The study depicted three basic scenarios. Group 1 corresponds to the telemedically assisted cohort, group 2 corresponds to the non-telemedically assisted cohort, and group 3 corresponds to a telemedically supported cohort for teaching purposes. The target case number of all three groups was 400 patients (20 calculated dropouts included). (3) **Discussion:** This study might help to clarify whether telemedicine-assisted ultrasound by a qualified expert is non-inferior to presence sonography concerning technical success and whether one of the interventions is superior in terms of efficacy and safety in one or more secondary endpoints. Randomization was provided, as every patient who needed an AU was included and then randomized to one of the groups. The third group consisted of a lower number of patients who were selected from group 1 or 2 for teaching purposes in case of rare diseases or findings. (4) **Conclusions:** The study investigates whether there are benefits of telemedical ultrasound for patients, medical staff, and the health care system.

## 1. Introduction

Complex diagnostic findings and interventions in abdominal ultrasound with the presence of an experienced examiner can currently only be offered in very few rural areas. In rural areas, the presence of experienced ultrasound specialists is often scarce; therefore, other more cost-intensive imaging techniques, e.g., computed tomography (CT), are performed. Furthermore, transfers of patients to higher-level-of-care hospitals are often required for further diagnostic examinations [1,2]. These transfers can be costly and time consuming as well as risky for patients and paramedical staff [3,4]. In these settings, the use of point-of-care ultrasound (POCUS) would be an opportunity, which has been investigated in previous studies, due to its safe and rapid bedside performance at low cost and with almost no learning curve needed for the investigator [5,6]. Sonographic imaging is not restricted to the abdomen but can depict many regions of the body. The use of POCUS has shown improvements in diagnosing findings and in shortening the time of management decisions in emergency care [7]. Therefore, training in POCUS as part of medical education has been recommended by several international medical associations for many years [8,9,10]. Recent studies have shown that tele-ultrasound POCUS courses during the COVID-19 pandemic have been as effective as the traditional in-person POCUS courses before [11]. POCUS offers benefits, including portability, real-time imaging, and safety compared to X-rays or CT scans. However, it is worthwhile to mention its limitations in terms of depth penetration, image quality, and the need for proper training in interpretation. The main question of our study investigates whether digitally supported sonographic assistance is equal to face-to-face assistance in a hospital’s daily routine. For this purpose, a telemedical connection of the Department for Endoscopy and Ultrasound of the Carl-Thiem-Klinikum (CTK), as a hospital at the secondary care level, to the Naemi-Wilke-Stift Guben, as a hospital at the primary care level, was tested in this study. In addition, the Charité-Universitätsmedizin Berlin (a University hospital at the tertiary care level) was included to provide an independent assessment of the telemedical ultrasound, as a supervisor of this telemedical support, for quality assurance.

The project aims to determine whether transfers to the CTK and thus the additional health costs and inconveniences for the patients caused by patient transport can be prevented. At the same time, specialized diagnostics and therapy can be carried out in the hospital for basic and standard care. In addition, as part of the project, professional training via digital application of the treating medical colleagues and the nursing staff in the hospital for basic and standard care through digital assistance in examinations carried out in the CTK was planned. For this purpose, the colleagues at the Naemi-Wilke-Stift Guben could participate telemedically in sonographic examinations in the CTK and thus expand and improve their expertise in sonographic diagnostics and interventions. Furthermore, the project offers the opportunity to discuss difficult findings directly with one another across disciplines and hospitals and to coordinate further diagnostic and therapeutic measures. The project thus offers not only the possibility of digital assistance for sonographic examinations and sonographically supported interventions but also the possibility of digitally supported further training for colleagues working in abdominal ultrasound. This means that numerous sonographic findings from patients were presented digitally and evaluated via a second opinion in a higher-level-of-care center. In addition, interesting sonographic findings could also be sent to train medical staff via telemedicine by a specialist in ultrasound. Therefore, even experienced examiners may benefit from the inter-colleague exchange and the discussion of rare findings due to different working fields, e.g., general internal medicine, surgery, or oncology. This was guaranteed by the possibility of a professional exchange with the Charité—Universitätsmedizin Berlin in the context of (telemedical) case reviews or the planning of further diagnostic steps such as special imaging or biopsies in complicated or rare cases. As a subsequent question, it was examined whether indications or certain patient clientele, possibly also gender-specific, emerged from the broad treatment cohorts (in principle, almost all patients with an indication for abdominal ultrasound) as disproportionately benefiting from a telemedical sonographic consultation.

## 2. Materials and Methods

This was a prospective, multicenter, randomized, open-label, and 2-arm non-inferiority study with a parallel group design. It included 200 patients per study arm, with a total number of 400 patients and with an anticipated (calculated) number of dropouts of 20 patients. Patients were recruited from 1 general hospital (n = 200 patients) and 1 higher-level-of-care hospital (n = 200 patients) in the state of Brandenburg, Germany. Each of the 2 study centers performed the ultrasound procedure with the best local expertise. Due to the local structure, a POCUS exam was conducted by the attending physician and not by a radiologist. The patients were selected and monitored according to the indication that they required abdominal ultrasound. Telemedically supported ultrasound and in-presence ultrasound with telemedical demonstration were included. The evaluating sonographers in the secondary and tertiary care centers were gastroenterologists with experience in abdominal ultrasound (>6000 sonographies). The study depicted three basic scenarios. The exposed group 1 corresponded to the telemedically assisted cohort (n = 200), the non-exposed group 2 corresponded to the non-telemedically assisted cohort (n = 200). Additionally, there was an exposed group 3 (n = 20), which corresponded to a telemedically supported cohort for teaching purposes. Patients of group 3 included those recruited in group 1 or 2 who needed an additional ultrasound for follow-up or control of uncertain findings during their hospitalization. Interesting sonographic findings could then be demonstrated by an expert in ultrasound to other medical staff using the same telemedical equipment as for group 1 and 2. The difference is that, in this case, the expert conducted the ultrasound, and the demonstration was transmitted to a broader medical staff of the hospital at the primary care level. To reduce selection bias, group 3 patients were recruited from groups 1 or 2, and an ultrasound was performed by the same examiner for teaching purposes only in case of rare or interesting findings. Therefore, group 3 patients received a follow-up exam conducted by the same examiner as the state-of-the art continuous medical education.

The technical requirements of the US devices at the respective study sites were functionally identical, even though US devices from different manufacturers were used, i.e., color doppler, as well as convex and linear, probes were available at all sites.

Furthermore, additional data were evaluated using certain questionnaires. An already established PDRQ-9 questionnaire [12,13] was adapted to evaluate the initial patient–physician relationship during the sonography or the telesonography. Furthermore, the questionnaire evaluated the communication during the telesonography between the doctors working in sonography. An established QLQ-C30 questionnaire [14] for the patients was also available for the evaluation of additional anamnestic data of the patients. This enabled drawing conclusions about the types of diseases that were more or less recognizable by sonography, which may significantly reduce the diagnostic period and lead to faster therapy. Additional patient data and information during the inpatient stay were also used to analyze the effects on further treatment measures. This included all sonographic findings and other imaging findings that were already available or occurred in the course of the hospital stay, especially from CT and magnetic resonance imaging (MRI). Furthermore, this included preliminary and leaving diagnoses, laboratory values, treatment data, and findings, including external findings that were entered into the hospital information system, medical history, and medication.

### 2.1. Primary Endpoint

The primary endpoint was savings in healthcare costs, defined as reduced personnel, resources, and additional diagnostics (such as radiological imaging MRI/CT) by faster therapy decision through ultrasound use.

### 2.2. Secondary Endpoints

The secondary endpoints were as follows:

Clinical success, defined as the reduction in the time to diagnostic finding with the use of ultrasound;

Duration of hospital stay in days;

Number of reduced referrals and transfers to a hospital with higher level of or priority care through successful telemedical assistance (especially from Guben [primary care level] to the CTK [secondary care level]);

Number of re-examinations from the day of the first examination to the end of the follow-up;

Subgroup analysis of the POCUS indication, defined as symptoms or suspected diagnosis;

Technical feasibility, defined as the successful and stable implementation of a diagnostic ultrasound reasonably useable in daily routine to allow for the remote telemedical assistance of an approved specialist in abdominal ultrasound.

### 2.3. Inclusion Criteria

The inclusion criteria were as follows:

Patients ≥ 18 years of age;

Indication for an abdominal ultrasound examination;

Written informed consent by the patient.

### 2.4. Exclusion Criteria

The exclusion criteria were as follows:

Patients participating in another study;

Patients who were unable to give written consent themselves.

### 2.5. Examinations

The non-telemedically assisted ultrasound at site was performed by a doctor with at least 1 year of experience performing bedside abdominal ultrasound, having completed over 100 abdominal ultrasounds but without the certification of expertise at a DEGUM level [15,16]. The telemedical assistance for an abdominal ultrasound was performed by a gastroenterologist, who was an expert at the DEGUM 2 level with experience of >10 years in performing clinical ultrasound, including more than 6000 sonographies in total.

### 2.6. Follow-Up

The study and the corresponding control visits ended for each patient after a follow-up of 1 month after the intervention. Each study center was obliged to document study patients who met the inclusion criteria but were not included in the study. Excluded patients and the reasons for exclusion are presented in an adapted CONSORT flow diagram [17].

### 2.7. Data Management

All data collected as part of the study were recorded on standardized questionnaires and documentation sheets. The investigators or study assistants delegated by them ensured the complete documentation of all patient data to be collected according to the study protocol. The evaluation of the questionnaires and further data collection were the responsibility of the study management in Guben and Cottbus. They were supported by the study center of the Carl-Thiem-Klinikum Cottbus and the research subsidiary of the Carl-Thiem-Klinikum, namely, the Thiem-Research GmbH. First of all, the cohorts were evaluated at the study sites themselves, and the results were sent to the other study sites in a pseudonymized form. After the end of the study, the data were initially stored in pseudonymized form and, if necessary, used for scientific publications. If patient data are published, this will be exclusively in an anonymous form. According to the medical profession regulations, all important study documents will be stored for at least 10 years after the end of the examination. The Director of Studies is responsible for the storage of the data. The documents should contain the protocol, ethics committee vote, patient information, declaration of consent as a template, and the signed copies in the original and final reports, questionnaires, and documentation. Investigators will store all study data (source data) including the patient identification list and relevant correspondence according to Section 4.9 of the ICH Good Clinical Practice Guideline (E6) [18]. Any change in the ownership of the data will be documented. All data, including patient identification, will be made available to regulatory authorities upon request.

### 2.8. Safety

Since the ultrasound is a gentle and radiation-free procedure with no adverse events expected, no extra Reporting Form for adverse events was necessary.

### 2.9. Statistical Analysis

This study is a purely descriptive proof-of-concept study, which for the first time included target values such as the length of time the patient is in bed, the implementation of further diagnostics such as CT, endoscopic retrograde cholangiopancreatography (ERCP), as well as the number of transfer transports that should be examined and may show a correlation to an added value of telesonographic examination. Our study is intended to provide initial indications as to whether telesonographic assistance could result in faster diagnosis (CT) or therapy (ERCP) and possibly a shorter patient stay in the hospital or reduced transfers to other hospitals. The sample size was defined as 200 patients in Guben and 200 patients in Cottbus with random assignments within these groups in advance. Further questions were intended to be examined more closely and statistically evaluated in a follow-up cohort. Group 3 patients were recruited from telemedical-assisted subjects within Groups 1 or 2 to provide a telemedicine demonstration of interesting sonographic findings to medical personnel. This was exclusively for the training and further education of medical staff in Guben and Cottbus. The data are evaluated in an electronic database (probably Microsoft Excel for Windows, Microsoft Office Suite 2016 Version 16.0 (2015), Microsoft Corp (Redmond, WA, USA) and/or IBM SPSS Statistics for Windows, Version 29.0. (2023), IBM Corp (Armonk, NY, USA). In the group comparison, we apply non-parametric tests to (un)dependent samples (Mann–Whitney U, Wilcoxon sign rank) and present the median with IQR. The chi-square test is used for comparisons between groups with categorical variables. A significance level of *p* < 0.05 is defined as statistically significant.

### 2.10. Status and Timeline of the Study

The first patient was enrolled on the 18th of January 2023. Total duration: 15 months, duration of clinical phase: 6 months, FSI (first subject in): 18 January 2023, LSI (last subject in: 31 July 2023, LSO (last subject out): 31 August 2023, DBL (database lock): 31 August 2023, completion of statistical analysis: 20 March 2024, completion of study report: 30 March 2024.

## 3. Discussion

The COVID-19 pandemic has increased the use of telemedicine in Germany, while recent studies in developing countries, e.g., India and Africa have already shown that telemedical consultations improve patient access to the healthcare system [19,20]. Furthermore, healthcare providers and healthcare centers in rural areas benefit from telemedicine through reduced healthcare costs, easy access to better medical expertise, and better access to patients in rural areas.

Further studies have shown a growing use of telemedicine during the COVID-19 pandemic in western countries using telematic consultations, too, promising a permanent role in future telematic postoperative follow-up [21]. Although studies have shown the positive benefit of telemedical consultations in general, to date, only few studies have examined POCUS in rural [22] or urban emergency settings [23,24,25]. The comparison between urban and rural need for further diagnostic imaging techniques, e.g., CT would be interesting to investigate [26] and is within the focus of this study for evaluation.

### 3.1. Strengths of This Study

Digital health techniques can reduce inequalities in access to health care and allow health care centers at different levels of care to co-operate for better and effective health care management of patients [20,27]. This study might help to clarify whether telemedicine-assisted ultrasound by a qualified expert is non-inferior to in-presence sonography (by a qualified expert) concerning clinical success. Furthermore, teaching purposes for medical staff are investigated, which may provide future opportunities in the field of telemedicine-assisted investigations. In addition, cost-effectiveness in terms of reduced transfer transports or further radiological imaging techniques (e.g., CT or MRI) in the case of unclear sonographic findings are being investigated and might show significant savings in healthcare costs and also an improvement in the quality of patient care burden for each individual patient. Ambulance transport and the corresponding waiting times as well as the monitoring of patients, some of whom are severely impaired, are to be regarded as labor-, time- and cost-intensive in this context. Appropriate telemedical sonographic assistance could lead to corresponding savings here. The use of POCUS offers several benefits in this study. It allows performing bedside real-time imaging that can help to make quick and accurate decisions regarding patient care and is considered to be a safer alternative to other imaging modalities, such as X-rays or CT scans, as it does not involve ionizing radiation. Furthermore, with sonographic assistance, for example, the interpretation and diagnosis of unclear masses, tumors, etc., could be improved, and repeated examinations or further diagnostics (such as computed tomography) could possibly be reduced or the indication for use might be made faster. This would save further resources in terms of personnel and equipment and would reduce the hospital stay of the patients through faster diagnosis established by a specialist. Randomization of the study arms might underline here another strength of this study, and finally, no specific education, e.g., in handheld POCUS is necessary due to the telemedical access to a sonographic specialist.

### 3.2. Limitations

Despite the benefits of POCUS in providing a valuable and quick sonographic assessment, it cannot replace more comprehensive imaging techniques like CT or MRI scans. Especially in obese patients or those with significant anatomical variations, POCUS is limited in terms of its depth penetration. In addition, the interpretation of POCUS, especially in the non-telemedically assisted cohort (Group 2) of our study requires expertise and the training of the healthcare professional. Due to the use of a sonographic specialist in the telemedically assisted cohort (Group 1), there could be some bias regarding the interpretation and detection rate. When using digital health care techniques, health inequities, e.g., digital and health illiteracy are often mentioned as weaknesses at the patient level, while lack of training in digital health care techniques is often mentioned as a weakness from the health care provider’s perspective [20]. In our study, the weakness of digital illiteracy might be found, e.g., in wrong understanding of the questionnaires especially by older patients, who might not ask if they do not understand the questions correctly because they might feel ashamed. A further typical limitation of a randomized trial is the poor external validity due to its design to provide evidence that the telemedicine-ultrasound does produce an effect in the study population. Due to the limited number of participants (n = 400), there is a lack of generalizability. Furthermore, only two hospitals were included due to its proof-of-concept design. This is also related to a low external validity because the study population may be unrepresentative [28].

## 4. Conclusions

Although recent studies may show a benefit of telemedicine in rural areas and in conditions like the COVID-19 pandemic when patients had less access to face-to-face consultations due to access routes or restrictions such as lockdowns, only a few studies have investigated the role of telemedical ultrasound in rural areas. The study therefore investigates whether there are benefits of telemedical ultrasound for patients, medical staff, and the health care system.

### 4.1. Dissemination Plans

The publication and presentation of the study results in an anonymous form is intended, and no other person except the researchers in this study and the members of the local Institutional Review Boards will have access to the clear data sets in accordance with the Law on the Protection of Data of a Personal Nature. The study results will first be communicated in a trusting manner to all participating study centers and then submitted to a peer-reviewed publication in a specialist journal. The author or authors of the publication must be sufficiently authorized by the study center to publish the study results. The use of gathered data for scientific publications is furthermore explicitly mentioned within the Informed Consent forms.

### 4.2. Amendments

Amendments to the protocol will be reported to all investigators, the local Institutional Review Board, all participants, and the journal.

### 4.3. Premature Termination of the Study

An interim analysis was performed when 100 patients were recruited in each study site. If an intervention was proven to be clearly beneficial or harmful compared to the concurrent control based on a pre-defined analysis of an incomplete data set while the study was on-going, the investigators could stop the study early. Every patient had the right to withdraw his or her consent to participate in this clinical trial at any time, without providing a reason. If possible, the time and reason for the termination of the study was recorded in the Case Report Forms (CRF). The data collected until then were also noted in the CRF. All investigators involved must be informed immediately of any stoppage or interruption in the clinical trial. The decision is binding among all study centers and investigators. If the clinical trial is terminated prematurely, all study materials (completed, partially completed) must be returned to data management.

## Data Availability

The metadata of this study DRKS00031803 are available upon reasonable request.
